# Roles of the Receptor for Advanced Glycation End Products and Its Ligands in the Pathogenesis of Alzheimer’s Disease

**DOI:** 10.3390/ijms26010403

**Published:** 2025-01-05

**Authors:** Wen Li, Qiuping Chen, Chengjie Peng, Dan Yang, Si Liu, Yanwen Lv, Langqi Jiang, Shijun Xu, Lihua Huang

**Affiliations:** 1 School of Pharmacy, Chengdu University of Traditional Chinese Medicine, Chengdu 611137, China; 15196681941@163.com (W.L.); m18383105971@163.com (Q.C.); 19224003161@163.com (C.P.); yangdan@stu.cdutcm.edu.cn (D.Y.); 13350714739@163.com (S.L.); 17380639946@163.com (Y.L.); jiang20001108@163.com (L.J.); 2 State Key Laboratory of Southwestern Chinese Medicine Resources, Chengdu 611137, China; 3 Institute of Material Medica Integration and Transformation for Brain Disorders, Chengdu University of Traditional Chinese Medicine, Chengdu 611137, China

**Keywords:** AD, RAGE, AGES, HMGB1, Aβ, S100

## Abstract

The Receptor for Advanced Glycation End Products (RAGE), part of the immunoglobulin superfamily, plays a significant role in various essential functions under both normal and pathological conditions, especially in the progression of Alzheimer’s disease (AD). RAGE engages with several damage-associated molecular patterns (DAMPs), including advanced glycation end products (AGEs), beta-amyloid peptide (Aβ), high mobility group box 1 (HMGB1), and S100 calcium-binding proteins. This interaction impairs the brain’s ability to clear Aβ, resulting in increased Aβ accumulation, neuronal injury, and mitochondrial dysfunction. This further promotes inflammatory responses and oxidative stress, ultimately leading to a range of age-related diseases. Given RAGE’s significant role in AD, inhibitors that target RAGE and its ligands hold promise as new strategies for treating AD, offering new possibilities for alleviating and treating this serious neurodegenerative disease. This article reviews the various pathogenic mechanisms of AD and summarizes the literature on the interaction between RAGE and its ligands in various AD-related pathological processes, with a particular focus on the evidence and mechanisms by which RAGE interactions with AGEs, HMGB1, Aβ, and S100 proteins induce cognitive impairment in AD. Furthermore, the article discusses the principles of action of RAGE inhibitors and inhibitors targeting RAGE-ligand interactions, along with relevant clinical trials.

## 1. Introduction

Alzheimer’s disease (AD) is the leading neurodegenerative disorder globally, characterized by a gradual onset. It is responsible for 60–70% of cases involving progressive cognitive decline among older adults [1]. It primarily manifests as progressive cognitive decline, including memory loss, diminished thinking and reasoning abilities, decreased judgment, and decision-making skills, and increasing difficulties in daily living, often accompanied by various psychiatric and behavioral symptoms. Initially, patients typically experience declines in memory and cognitive functions; as the disease progresses, they may not only forget their loved ones but also undergo significant changes in personality and behavior [2].

The receptor for advanced glycation end products (RAGE) is a protein that spans the cell membrane consisting of 404 amino acids [3] and is capable of binding multiple ligands. It is predominantly located in vascular smooth muscle cells, endothelial cells, and cardiomyocytes. In most adult tissues, RAGE is typically present at low levels; however, its expression increases significantly in the arterial walls in areas affected by vascular disease. Research indicates that RAGE is widely distributed across different cells and tissues, exhibiting constitutive expression at different embryonic developmental stages. However, in adults, RAGE expression levels are typically low and gradually increase with aging [4]. RAGE has a dual role: at low concentrations, it can have beneficial effects in some cells, such as activating neuronal differentiation, enhancing the resistance of neurons and muscle tissues to apoptotic stimuli, and eliminating excess myocytes through apoptosis. At high concentrations, RAGE may have harmful effects, often manifested as increased inflammatory responses and delayed neuronal regeneration and tissue repair.

Epidemiological studies and imaging data increasingly emphasize RAGE’s role in facilitating neurodegenerative changes associated with AD. Lue et al. [5] found that in deceased AD patients, RAGE levels were significantly elevated in the hippocampus and inferior frontal cortex. Additionally, a positive correlation existed between RAGE levels and the severity of brain pathology. The heightened expression of RAGE on the membranes of neurons and microglia is associated with the mechanisms of neuronal dysfunction and death in AD. Inhibiting RAGE signaling in microglia can alleviate pathological conditions in AD mouse models by restoring some synaptic plasticity. Supporting this perspective, research has shown that introducing mesenchymal cells capable of secreting RAGE into AD mouse models can prevent neuronal loss [6]. Research indicates that neuron-specific overexpression of RAGE exacerbates the pathological changes in J20 transgenic mice, while reduced RAGE expression decreases amyloid pathology and improves cognitive dysfunction in J20 mice [7,8]. RAGE promotes neuroinflammation in response to the buildup of Aβ, and increased levels of RAGE or knockout of RAGE signaling impair RAGE activity, thereby improving cerebral blood flow, slowing cognitive decline, and alleviating neuroinflammation [9]. Therefore, targeting RAGE may present a promising treatment avenue for AD.

RAGE can bind and mediate cellular responses to various damage-associated molecular patterns (DAMPs), such as advanced glycation end products (AGEs), beta-amyloid peptide (Aβ), high mobility group box 1 (HMGB1), S100 calcium-binding proteins, and amphoteric proteins. Given the critical role of RAGE in AD and its multi-ligand characteristics, we explored how interactions between RAGE and its ligands, such as AGEs, Aβ, HMGB1, and S100, influence the pathogenesis of AD. Additionally, we discussed the potential of inhibitors targeting RAGE–ligand interactions in AD therapy, providing a theoretical foundation for the development of targeted therapeutic strategies.

## 2. Pathogenesis of Alzheimer’s Disease

Beta-amyloid peptide (Aβ) is a low-solubility variant composed of 42 amino acids, often simply called Aβ. At very low concentrations, Aβ may stimulate neurite growth, provide neuroprotection, and even exhibit antimicrobial properties [10]. However, when Aβ is produced in excess, it forms amyloid cores in typical plaques, which are among the defining pathological characteristics of AD. Studies have indicated [11] that once Aβ appears in the cytoplasm, it may form pathological aggregates similar to perinuclear aggregates. Amyloid precursor protein (APP) is a glycosylated protein predominantly located in cell membranes, with the highest concentration occurring in the brain. Research suggests that APP, when bound to the membrane, serves as a copper chaperone, providing cellular protective functions. APP is cleaved by three intracellular enzymes—alpha, beta, and gamma secretases—into various peptides, with its soluble cleavage products potentially having protective effects as well [12]. For instance, the soluble form of APP derived from alpha-secretase (APPsα) exhibits neuroprotective and neurotrophic activities [13], while another secreted APP variant, APPsβ, exerts biological activity by modulating certain metabolic pathways [14], though its neuroprotective effects are not as pronounced as those of APPsα. The primary component of Aβ is produced by the abnormal degradation of APP in the neuronal cytoplasm [15]. The abnormal cleavage of APP culminates in the production of Aβ, along with a fragment referred to as the APP intracellular domain (AICD). Subsequent fragments primarily exhibit cytotoxic effects. Under normal physiological conditions, The ubiquitin–proteasome system (UPS) primarily degrades AICD and Aβ. However, in older adults, insufficient UPS activity may enhance the accumulation of insoluble cytoplasmic aggregates.

Most AD patients exhibit sporadic forms, with hereditary AD accounting for less than 2% of diagnosed instances [16]. Notably, nearly 25% of sporadic AD patients carry the Apolipoprotein E4 (ApoE4) allele located on chromosome 19. While the exact mechanisms through which the ApoE4 allele influences AD and alters Aβ levels are not yet fully understood, studies investigating the in vitro removal of Aβ from the brain into peripheral circulation indicate that this process may be influenced by different subtypes of Apolipoprotein E (ApoE). Individuals carrying the APOE4 allele demonstrate diminished elimination of Aβ from the brain. Additionally, the APOE4 allele appears to be linked to telomere shortening and a faster rate of aging. Cholesterol is an important molecule in brain physiology, and changes in its levels may lead to abnormal Aβ metabolism, ultimately triggering neurodegenerative changes. Cholesterol is transported into neurons through ApoE and low-density lipoprotein receptor-related proteins 1 and 8 (LRP1 and LRP8). It is believed that the development of AD may be linked to changes in the function of LRP1 or LRP8 receptors, which could result in inadequate cholesterol supply to neurons and poor metabolism of the copper chaperone APP [17]. Additionally, the crucial trace element selenium (Se) is transported to the brain via selenoprotein P, with LRP8 receptors playing a role in this process. Furthermore, LRP1 is involved in the transport of Aβ from the brain to the peripheral bloodstream. Alongside these factors, comorbidities such as elderly hypertension, metabolic syndrome (MetS), and dyslipidemia are also considered risk factors for sporadic AD.

Studies have found that disruption of the blood–brain barrier (BBB) can lead to early cognitive impairment. BBB dysfunction has been observed as an early sign of AD, even before the onset of dementia or neurodegeneration [18]. Researchers characterized the permeability of the blood–brain barrier (Ktrans BBB) in the hippocampus and its subregions using dynamic contrast-enhanced magnetic resonance imaging (DCE-MRI) along with post-processing analysis. The initial disruption of the blood–brain barrier occurs in the hippocampus—a region closely linked to learning and memory functions—during normal aging and in cases associated with mild cognitive impairment (MCI) [19].

Mitochondria are highly dynamic and complex organelles within cells, primarily responsible for energy production, while also playing crucial roles in processes such as cell death, signal transduction, apoptosis, reactive oxygen species (ROS) generation, and calcium homeostasis. These processes are often disrupted in neurodegenerative diseases. Neurons are highly dependent on mitochondria, which are vulnerable to dysfunction due to their complex morphology and high metabolic demands. The loss of neuronal function is a hallmark of neurodegenerative diseases, making it closely linked to mitochondrial dysfunction [20]. In the brain tissue of AD patients and AD animal models, evidence of decreased brain metabolism is commonly observed, indicating impaired glucose metabolism and mitochondrial dysfunction [21]. There is evidence that the interaction between mitochondria and the endoplasmic reticulum (ER) plays a key role in the pathogenesis of AD. Mitochondria-associated ER membranes (MAMs) regulate various processes at the interface between mitochondria and the ER, including but not limited to signal transduction, calcium homeostasis, phospholipid synthesis, and trafficking. These functions are disrupted in AD [22]. C99 (the direct precursor of Aβ) is present in MAMs, and the accumulation of C99 may mediate the increased MAM activity and subsequent mitochondrial dysfunction. The increase in C99 leads to enhanced production of ceramide, which is an inhibitor of mitochondrial respiration and a pro-apoptotic molecule. MAM dysfunction may indirectly disrupt mitochondrial processes, and the increased ER–mitochondrial contact through MAMs could explain phenomena such as increased calcium transport and altered phospholipid profiles observed in AD. Research suggests that the formation of abnormal mitochondrial–endoplasmic reticulum contact sites (MERCS) in AD may lead to mitochondrial dysfunction. In MERCS [23], autophagosomes form in conjunction with Aβ plaques [24], while Ca^2+^ flows from the endoplasmic reticulum into the mitochondria, and Aβ aggregates obstruct the export of mitochondrial Ca^2+^. Aβ has also been shown to form calcium-permeable pores on the plasma membrane, allowing calcium to accumulate in the cytoplasm, leading to excessive calcium uptake by the mitochondria, which subsequently generates free radicals and causes mitochondrial dysfunction [25].

Oxidative reactions are an essential foundation for constructing the intricate networks of physical and chemical processes associated with life. These reactions are integral to aerobic metabolism, the synthesis of both small and large molecules, signaling within and between cells, and the defense mechanisms of immune cells [26]. However, excessive and prolonged oxidative phenomena are associated with cellular damage and destruction, which can trigger various inflammatory, tumor, and degenerative diseases at the organism level [27]. Oxidative stress plays a role in the pathophysiology of various diseases, such as atherosclerosis, AD, and diabetes [28]. In patients with AD, peripheral tissues often exhibit elevated levels of lipid peroxidation and a diminished capacity for both enzymatic and non-enzymatic antioxidants. Highly ROS interact with unsaturated fatty acids, resulting in the formation of lipid peroxides such as malondialdehyde (MDA) and 8, 12-iso-iPF2α [29], which can damage DNA, lipids, and proteins [30]. Research has demonstrated that the nervous system is especially vulnerable to ROS-induced damage. Neuronal cell death is linked to ROS-induced changes, which include modifications in membrane fluidity, rigidity, permeability, and the transport of lipid molecules within neurons. Furthermore, numerous studies indicate that reactive oxygen species—such as superoxide anions, hydrogen peroxide, and hydroxyl radicals—along with nitric oxide, play a significant role in the neurodegenerative processes linked to AD [31,32,33]. Neuronal injury can induce microglial activation, which in turn causes excessive generation of superoxide anions and ROS. This process also involves the interplay between cytokines and nitric oxide, ultimately culminating in oxidative stress.

Neuroinflammation arises from the abnormal activation of immunological cells within the central nervous system (CNS), frequently due to CNS injury, infection, or autoimmune conditions. This process can have both positive and negative impacts on neurodevelopment and associated mechanisms. The inflammatory response, marked by the secretion of cytokines and growth factors during temporary activation, has a beneficial effect on tissue development and recovery following injury. Nevertheless, persistent or uncontrolled inflammatory responses may contribute to the emergence of several neurodegenerative disorders, such as AD, Parkinson’s disease (PD), amyotrophic lateral sclerosis (ALS), and multiple sclerosis [34]. Previous research overwhelmingly indicates that neuroinflammation has a key role in the initiation of AD, particularly in hippocampal neurons [35]. This is evidenced by elevated levels of various inflammatory mediators, including nuclear factor-kappa B (NF-κB), tumor necrosis factor-alpha (TNF-α), interleukin-1β (IL-1β), and interleukin-6 (IL-6) in individuals with AD [36]. Pro-inflammatory cytokines such as IL-1β, IL-6, and TNF-α can disrupt the functioning of hippocampal neurons. This impairment affects processes like long-term potentiation (LTP) and dendritic branching, both of which are vital for learning and memory [37].

## 3. RAGE

### 3.1. RAGE Structure

RAGE is characterized by a hydrophobic domain that spans the membrane, a charged-residue-rich cytoplasmic tail, and an extracellular district. The extracellular portion contains an N-terminal V-type immunoglobulin domain along with two C-type immunoglobulin domains, referred to as C1 and C2. This is succeeded by a membrane-embedded domain and a cytoplasmic domain that is notably rich in charged residues. A versatile linker distinguishes the self-sufficient C2 zone from the structural unit created by the V-type and C1 domains. The variable V zone is composed of two β-sheets that are linked by disulfide bridges. Meanwhile, hydrogen bonds and hydrophobic interactions connect the V and C1 regions, creating a unified structural unit. The molecular exterior of the VC1 domain displays hydrophobic pockets alongside regions with a positive charge [38,39]. Crystallographic studies of the extracellular domain, along with NMR spectroscopy of the ligand-binding domain, indicate that the V domain of RAGE possesses a hydrophobic cavity and a flexible region composed of residues 55 to 71. This flexible segment enhances the adaptability of the hydrophobic pocket, allowing RAGE to engage productively with ligands. Consequently, the V-type region is considered the primary location for interactions between RAGE and potential extracellular ligands.

**Figure 1:**
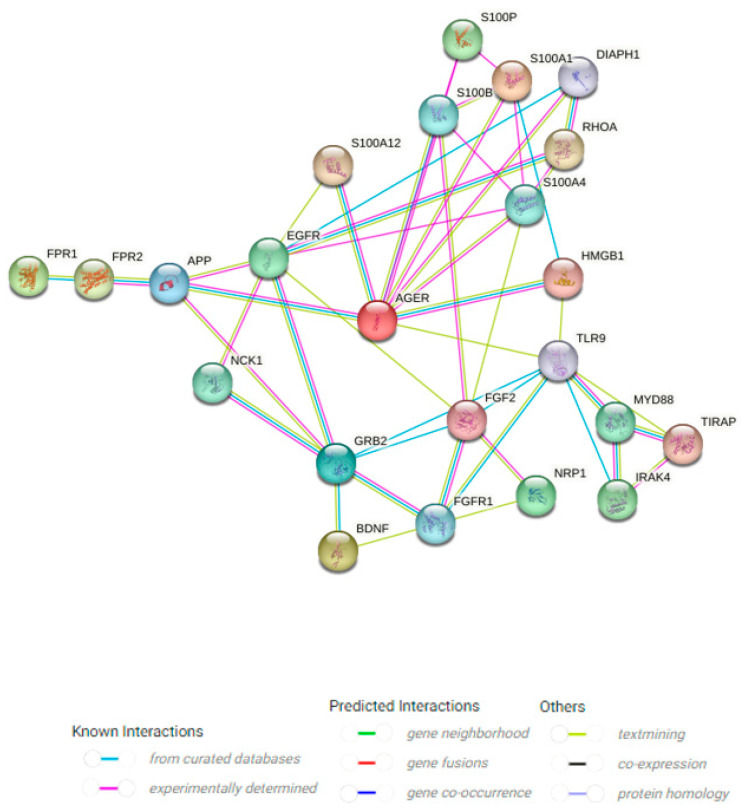
String representation of RAGE–ligand interactions. To ensure understanding, this representation includes exclusively the most significant and extensively researched protein interactions. The highlighted interactions consist of AGER (RAGE), HMGB1, the S100 family of calcium-binding proteins (S100B, S100P, S100A1, S100A4, S100A12), DIAPH1, RhoA, TLR9, MyD88, TIRP4, IRAK4, EGFR, GR-B2, FGFR1, BDNF, FGF2, NRP1, NCK1, Aβ, FPR1, and FPR2. Abbreviations: RAGE, the receptor for advanced glycation end products; HMGB1, high mobility group box 1; DIAPH1, protein diaphanous homolog 1; RhoA, ras homolog family member A; TLR9, toll-like receptor 9; MyD88, myeloid differentiation primary response 88; TIRP4, toll/interleukin-1 receptor domain-containing protein 4; IRAK4, interleukin-1 receptor-associated kinase 4; EGFR, epidermal growth factor receptor; GR-B2, glucocorticoid receptor B2; FGFR1, fibroblast growth factor receptor 1; BDNF, brain-derived neurotrophic factor; FGF2, fibroblast growth factor 2; NRP1, neuropilin-1; NCK1, non-catalytic region of tyrosine kinase adaptor protein 1; Aβ, beta-amyloid peptide; FPR1, fMet-Leu-Phe receptor; FPR2, n-formyl peptide receptor 2. Source: https://string-db.org/ (Accessed on 30 August 2024).

Because RAGE identifies spatial structures instead of specific peptide sequences, it is efficient at binding to a range of chemical messengers that do not share sequence homologies. This property enables this versatile ligand receptor to be classified as a pattern recognition receptor (PRR). RAGE binds to various DAMPs and facilitates cellular responses. Beyond the initially identified ligand—AGEs—RAGE also collaborates with several other molecules, encompassing Aβ, HMGB1, S100 proteins (S100B, S100P, S100A1, S100A4, S100A12), protein diaphanous homolog 1 (DIAPH1), brain-derived neurotrophic factor (BDNF), epidermal growth factor receptor (EGFR), myeloid differentiation primary response protein MyD88, transforming protein ras homolog family member A (RhoA), toll/interleukin-1 receptor domain-containing adapter protein, and toll-like receptor 9 (TLR9). It is also predicted to bind to cytoplasmic protein non-catalytic region of tyrosine kinase adaptor protein 1 (NCK1), chromosome 3 open reading frame 52 (C3orf52), and Mitogen-Activated Protein Kinase 5 (MAPK5)—data drawn from Uniprot (https://www.uniprot.org/) and the Hit Prediction Database (http://www.hitpredict.org/) (Accessed on 30 August 2024) (Figure 1).

### 3.2. RAGE Isoforms

**Figure 2:**
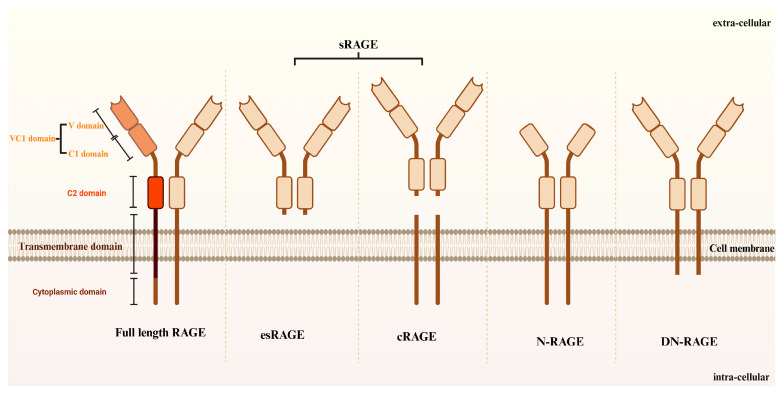
Structure and isoforms of RAGE. As part of the immunoglobulin superfamily, RAGE is categorized among cell surface proteins. It is a type I membrane protein that spans the membrane once and features one variable (V) sector along with two constant (C1 and C2) immunoglobulin-like domains in its external cellular environment. Following this is a transmembrane region, along with a cytoplasmic domain that has a high charge density. The primary isoforms of RAGE comprise full-length RAGE, cleaved RAGE (cRAGE), endogenous secretory RAGE (esRAGE), N-terminal truncated RAGE (N-RAGE), and dominant-negative RAGE (DN-RAGE) (Created in https://BioRender.com) (Accessed 27 September 2024).

In addition to the full-length RAGE, several isoforms exist, including cleaved RAGE (cRAGE) and endogenous secretory RAGE (esRAGE), which is a splice variant truncated at the C-terminus, lacking both the transmembrane and cytoplasmic domains. Other isoforms include N-RAGE (also known as RAGE splice variant 2), which is trimmed at the N-terminus and omits the V domain, and DN-RAGE (dominant-negative RAGE), which is characterized by the absence of the cytoplasmic domain [40] (Figure 2). sRAGE consists of both cRAGE and esRAGE. cRAGE is generated by the proteolytic cleavage of full-length RAGE, whereas esRAGE is produced through the splicing of RAGE mRNA. These two soluble RAGE isoforms can be differentiated by a distinct C-terminal sequence found in esRAGE. sRAGE functions as a mimic for RAGE ligands [41], binding to them without triggering intracellular signaling. Research suggests that the plasma concentrations of sRAGE are diminished in patients with AD relative to those in the untreated group. The stressors affecting the AGE-RAGE axis consist of AGE and RAGE, whereas protective factors include enzymatic degradation products of AGE, such as glyoxalase-1 and glyoxalase-2. Additionally, degradation products influenced by AGE receptors (AGER1 and AGER2), sRAGE, and substances that reduce AGE levels in the bloodstream also contribute to protection. Thus, sRAGE has a cytoprotective role in the interaction between AGE and RAGE. Clinical research indicates that elevated plasma concentrations of sRAGE are linked to a lower risk of several diseases, including AD.

### 3.3. RAGE Downstream Signaling Pathway

RAGE is recognized as a promoter of inflammation and oxidative stress. The interactions activate various signaling pathways, including ras-extracellular signal-regulated kinase 1/2 (Ras-ERK1/2), nicotinamide adenine dinucleotide phosphate (NADPH) oxidase, stress-activated protein kinase/c-Jun N-terminal kinase (JNK), renin-angiotensin system (RAS), Src kinase, p38 mitogen-activated protein kinase (p38 MAPK), phosphoinositide 3-kinase/protein kinase B (PI3K/Akt), Rho GTPase Cdc42/Rac, RhoA-associated kinase, protein kinase C beta II, and glycogen synthase kinase 3β (GSK-3β). These signaling pathways subsequently activate transcription factors downstream, including NF-κB, early growth response factor-1 (EGR-1), and specificity protein 1 (SP-1), thereby inducing the expression of various pro-inflammatory genes, such as vascular cell adhesion molecule-1 (VCAM-1), IL-6, TNF-α, and additional immune modulators [42,43].

RAGE is highly expressed across various cell types, such as endothelial cells, neurons, cardiomyocytes, mesangial cells, and immune cells. In neuronal cells, RAGE interacts with its ligands, triggering downstream activation of the mitogen-activated protein kinase (MAPK) family associated with Toll-like receptor 4 (TLR4), including p38 MAPK, ERK1/2, and JNK. Moreover, RAGE facilitates the movement of NF-κB into the nucleus, leading to the activation of various transcriptional target genes that promote inflammatory responses and cell death. The intracellular portion of RAGE is crucial for signal propagation and engagement of NF-κB. TLR4 is essential in the inflammatory response, facilitating two varied signaling pathways: MyD88-dependent and MyD88-independent pathways. Stimulation of the latter can result in the phosphorylation of inhibitor of kappa B kinase (IKK) and MAPK, thereby initiating signal transduction through both pathways. The first is the NF-κB signaling pathway. In the CNS, NF-κB is vital for modulating both hippocampal neurogenesis and neurodegeneration. Upon the initiation of TLR4, NF-κB emerges as the primary transcription factor involved in inflammation, activating upstream inhibitory proteins such as IKK and facilitating the phosphorylation of IκB. The second pathway involves the activation protein-1 (AP-1) pathway, in which upstream MAPK signaling governs the induction and nuclear relocation of ERK, JNK, and p38. This process further stimulates the nuclear transcription factor AP-1, which directly regulates the secretion of inflammatory agents like IL-1β, IL-6, and TNF-α. Some researchers have found [44] that the commencement of the TLR4 signaling cascade can culminate in neuronal degeneration and loss, thereby accelerating the progression of AD. Consequently, inhibiting the AP-1 and NF-κB signaling pathways may be crucial in the management of AD [45].

### 3.4. RAGE Inhibitors

The binding of RAGE to its ligands results in the generation of ROS and other inflammatory mediators that contribute to neuronal damage. Additionally, ROS can enhance the synthesis of ligand proteins. Therefore, RAGE antagonists may offer therapeutic benefits for treating AD. There are three main ways to block RAGE activity: inhibiting RAGE expression, blocking ligand binding, and inhibiting RAGE signaling [2]. Currently, most potential RAGE antagonists target the extracellular V domain [46], which is capable of binding to RAGE ligands (including AGEs and Aβ) or modulating RAGE signaling and downstream events.

FDA-approved Thiazolidinediones (TZDs) used for diabetes management diminish the AGE–RAGE interaction via an intermediary mechanism. They achieve this by inhibiting RAGE formation and promoting the manifestation of sRAGE via the implementation of peroxisome proliferator-activated receptor gamma (PPAR-γ). Recent studies suggest that thiazolidinediones can activate brain pathways regulated by Insulin-like Growth Factor 1 (IGF-1) [47]. Some clinical trials have evaluated the efficacy of TZD drugs in AD. For example, both rosiglitazone and pioglitazone, two commonly used TZDs, showed potential for improving cognitive function in early clinical studies. However, the results of these trials have been inconsistent [47], with rosiglitazone potentially posing serious side effect risks, and some studies failing to demonstrate significant clinical benefits. Therefore, the role of TZDs in the treatment of AD still requires further validation and optimization.

Azeliragon (PF-04494700), also referred to as TTP488 or TTP400022, is an antagonist of RAGE that competes for binding to its extracellular domain. It exhibits a high affinity for RAGE ligands, including AGEs, HMGB1, and S100B. Additionally, azeliragon has been shown to decrease levels of Aβ_1–42_. In transgenic mice with AD plaque development, daily administration of azeliragon (10 mg/kg via intraperitoneal injection or 20 mg/kg orally) significantly reduced inflammatory markers (TNF-α, TGF-β, and IL-1), amyloid deposition in the central nervous system, and improved cognitive function [48]. In a systemic amyloidosis mouse model, azeliragon reduced the accumulation of Aβ peptides in the spleen as well as the expression of IL-6 and macrophage colony-stimulating factor [49]. The safety and efficacy of azeliragon have been studied in Phase I and Phase II clinical trials in humans [48,49,50]. In the double-blind Phase IIa trial, patients with mild to moderate AD were administered high-dose azeliragon (27 patients, 60 mg/day for 6 days, then 20 mg/day for 10 weeks), low-dose azeliragon (28 patients, 30 mg/day for 6 days, then 10 mg/day for 10 weeks), or a placebo (12 patients). These doses appeared to be safe and well-tolerated, but the treatment showed no differences in plasma Aβ levels, inflammatory biomarkers, or cognitive measurements [49]. A larger multicenter study involving 399 AD patients (Phase II trial, NCT00566397) aimed to determine whether there was any evidence suggesting that azeliragon could slow the decline in cognitive abilities. A total of 135 patients were assigned to the high-dose azeliragon group (60 mg/day for 6 days, then 20 mg/day for 6 months), 135 patients to the low-dose group (20 mg/day for 6 days, then 5 mg/day for 18 months), and 132 participants to the placebo group [48]. In contrast, patients receiving the lower dose treatment showed a beneficial effect on the decline in cognitive abilities. While a phase II clinical trial [51] demonstrated that azeliragon reduced neuroinflammation by decreasing Aβ_1–42_ values, a phase III trial evaluating its efficacy and tolerability in individuals with slight AD was halted. Currently, other phase III trials of azeliragon are underway for individuals with mild AD and glucose intolerance.

FPS-ZM1 (4-chloro-N-cyclohexyl-N-(phenylmethyl)benzamide) is another antagonist of RAGE that attaches to the extracellular domain of the receptor. It inhibits oxidative stress induced by AGE in primary rat microglia. FPS-ZM1 suppresses the expression of NF-κB and downstream inflammatory mediators such as TNF-α, IL-1β, cyclooxygenase-2 (COX-2), and inducible nitric oxide synthase (iNOS). It also reduces the construction of NADPH oxidase (NOX), which is responsible for activating ROS production. FPS-ZM1 boosts the activity of antioxidant enzymes, including heme oxygenase-1 (HO-1), and helps reduce kidney damage in hypertensive rats. It also mitigates AGE-induced inflammatory responses in the hippocampus of these animals. Administering specific antagonists like TTP488 or FPS-ZM1 decreases neuroinflammation and amyloid accumulation while enhancing behavioral performance in mouse models of Alzheimer’s disease [48,52]. Although FPS-ZM1 has shown some potential in preclinical studies, translating it from laboratory research to clinical application still faces many challenges. For example, issues such as the side effects of FPS-ZM1, its pharmacokinetics, and how to effectively target neuroimmune cells in the brain require further investigation.

Inhibiting RAGE signaling can be achieved by drug formulations or exogenous administration of sRAGE to elevate its levels. Drug formulations, including pitavastatin and pravastatin, can increase serum sRAGE levels in humans; atorvastatin, fuvastatin, and lovastatin [53] increase sRAGE levels in isolated cell lines; atorvastatin [54] enhances both sRAGE and esRAGE levels in vitro, as do angiotensin-converting enzyme inhibitors (ramipril, perindopril) and the anti-diabetic drug rosiglitazone [55].

## 4. RAGE Ligands and Their Role in Neurodegenerative Diseases

### 4.1. AGEs

AGEs are a class of entities produced by the reaction between carbonyl compounds in carbohydrates and free amino groups in amino acids, a process collectively referred to as the Maillard reaction. The initial stage of this reaction occurs during thermal processing, when the nitrogen moiety of lysine or arginine residues combines with the carbonyl group of lessening sugars, resulting in the formation of Schiff base adducts. Following this, these Schiff bases rearrange to form a stable intermediate called the Amadori product. The Amadori intermediate can further interact with diverse other molecules through processes like fragmentation, oxidation, enolization, dehydration, and cyclization. This ultimately leads to the formation of various advanced products, including pigments, aromatics, and AGEs. Under conditions of prolonged storage or high-temperature degradation, Amadori intermediates can transform into α-dicarbonyl compounds. These compounds then irreversibly cross-react with the arginine and lysine residues of proteins during the final phases of the Maillard reaction, resulting in the assembly of AGEs. These reactions are particularly damaging because they can alter the structure of endogenous proteins and may also impact their function.

Endogenous AGEs are frequently associated with age-related and diabetes-related conditions. In both acute and chronic inflammatory processes, the activation of myeloperoxidase in neutrophils results in the manufacture of AGEs, which leads to the synthesis of the highly reactive glyoxal (GAD). Moreover, the oxidative process affecting particular amino acids, polyunsaturated lipids, and ketone bodies leads to an elevated generation of glyoxal, ultimately resulting in AGE production. In cases of chronic kidney disease (CKD) [56], AGEs arise from a diminished clearance of precursors involved in glycation reactions, as well as a reduction in antioxidant enzyme activity. Elevated levels of circulating glucose encourage the production of AGEs, and the internal effects of high blood sugar were initially noted in diabetic patients, who exhibited significant hemoglobin glycation. The connection between blood glucose levels and AGEs was discovered within the serum samples from individuals with diabetes. AGEs play a significant role in clinical research due to their association with oxidative stress and inflammation—processes that ultimately contribute to a variety of chronic illnesses, including cardiovascular diseases, diabetes, CKD, and neurodegenerative disorders. Importantly, AGEs promote oxidative stress, which in turn also facilitates the formation of AGEs. This indicates that AGEs can be generated even in the absence of elevated blood sugar levels, driven by oxidative stress.

Cooking techniques like roasting, grilling, and frying can considerably raise the levels of AGEs within foods. Food manufacturers frequently utilize these techniques to enhance the flavor, visual appeal, and aroma of their products. Carboxymethyllysine (CML) and methylglyoxal (MG) are recognized derivatives resulting from interactions between sugars and lipids or proteins, and they act as indicators of AGEs [57]. Research has indicated that individuals consuming high-protein diets, even if relatively healthy, exhibit increased serum levels of CML. Throughout the years, numerous databases have utilized CML as a supplementary measure to describe the AGE levels in different food categories, especially in those typical of the Western diet. Studies indicate that CML levels are highest in dry foods like bread, cereals, and cookies, as well as in dairy items, meats, and nuts, or in grain products subjected to high-temperature processing. In contrast, CML content tends to be lower in butter, fruits, vegetables, and coffee.

AGEs and their precursors, including MG, have the potential to modify the structure and activity of endogenous proteins, resulting in cellular impairment and tissue and organ damage. AGEs induce pathology primarily through two mechanisms. First, AGEs have the ability to cross-link proteins, which directly modifies their structure, characteristics, and functions. Second, AGEs initiate intracellular signaling via various receptor- and non-receptor-mediated pathways, resulting in an enhanced production of ROS and inflammatory cytokines. AGEs are broken down by enzymes and receptors in the body and are then eliminated through the kidneys. The primary degrading enzymes involved are glyoxalase I and II. The advanced glycation end product receptor 1 (AGER1) serves as a receptor that attaches to, internalizes, and degrades AGEs.

#### 4.1.1. Level of AGEs in AD

Research over the past 30 years has indicated that AGEs are associated with aging and neurodegenerative diseases [48,58]. During the aging process, AGEs progressively develop and accumulate in the body, which is a normal phenomenon. However, this process is accelerated in diabetes, neurodegeneration, and cardiovascular diseases [59].

Research has indicated that CML is present in the cytoplasm of neurons, astrocytes, and microglia within the brains of older individuals and those with Alzheimer’s disease [60]. An increase in AGE-positive cells has been observed in the neurons and astrocytes of individuals with Alzheimer’s disease. Studies have also demonstrated that AGEs are present in the senile plaques associated with AD, and CML co-localizes with tau protein in these individuals. Immunohistochemical techniques have identified the presence of AGEs in both senile plaques and neurofibrillary tangles (NFTs) in individuals with AD [61]. CML and Amadori product levels are increased in the cerebrospinal fluid of individuals with AD [62]. Elevated levels of AGEs in the skin of AD patients are linked to reductions in functional activity [63]. Spauwen et al. [64] reported elevated skin AGE autofluorescence in diabetic patients, which is associated with cognitive decline. Oxidative stress contributes to the formation of AGEs, which in turn serve as a source of superoxide anions. Research has shown that treating astrocytes with AGE-BSA increases the production of TNF-α and IL-1β, along with oxidative stress and nitric oxide release [65]. The reduction in BDNF levels caused by diabetes-induced hyperglycemia leads to damage to cerebral microvessels, also mediated by AGEs [66]. In an AD mouse model, intracranial injection of AGEs has exacerbated many pathological features of AD [67]. In conclusion, elevated levels of AGEs have been observed in the brain tissue, cerebrospinal fluid, plasma, and skin of individuals with AD, indicating a significant relationship between AGE levels and the disease’s pathogenesis.

#### 4.1.2. AGE-RAGE Axis and Its Downstream Signaling Pathways

The AGE-RAGE pathway is involved in the initiation and advancement of several chronic illnesses and metabolic syndromes. The degree of AGE formation is linked to the advancement of various related conditions, including cancer, diabetes, diabetic retinopathy and neuropathy, diabetic osteoporosis, atherosclerosis, heart failure, AD, ALS, PD, later stages of traumatic brain injury (TBI), and chronic obstructive pulmonary disease (COPD).

A number of in vitro investigations have shown that heightened activation of the AGE-RAGE axis contributes to the pathological processes associated with AD. In rodent studies, the administration of corticosterone (CORT) results in disturbances to circadian rhythms, heightened oxidative damage and inflammation, diminished quantities of neurotrophic factors like BDNF, and a reduction in hippocampal neurogenesis. These factors are believed to have a significant impact on the onset of depression. Jun Huang et al. [68] found that dihydromyricetin (DHM) decreased the levels of AGEs, RAGE, and pro-inflammatory factors in the murine hippocampus treated with CORT. This suggests that DHM may inactivate the AGE-RAGE signaling pathway. P-coumaric acid (p-CA) has been shown to reduce depressive-like behaviors and memory deficits induced by CORT. Its protective effects are thought to involve multiple targets and signaling pathways, with the AGE-RAGE pathway likely playing a central role. CORT activates the AGE-RAGE signaling pathway, whereas p-CA mitigates this action, highlighting its potential as a therapeutic agent for depression [69]. Sulforaphane (SFN) is a promising candidate for addressing toxicity associated with AGEs. Research has demonstrated a significant interaction between nuclear factor erythroid 2-related factor 2 (Nrf-2) and NF-κB, with Nrf-2 serving to negatively modulate the pro-inflammatory pathways driven by NF-κB [70]. SFN plays a crucial role in activating Nrf-2, which in turn inhibits NF-κB signaling pathways. SFN markedly diminishes the rise in RAGE expression caused by MGO-AGE in microglia, indicating that the inhibition of RAGE by SFN is essential for mitigating downstream inflammatory responses associated with the AGE-RAGE pathway. This discovery reinforces the role of SFN in reducing AGE-RAGE expression in pericytes and in downregulating the inflammatory cascade in both pericytes and endothelial cells [71].

The engagement of AGEs with RAGE on the cell membrane negatively impacts cellular functions. The AGE-RAGE axis can activate NF-κB, AP-1, cyclic AMP response element-binding (CREB) proteins, early growth response factor-1 (EGR-1), and signal transducer and activator of transcription 3 (STAT3), resulting in cell death across various types, such as neurons, endothelial cells, lung cells, and muscle cells [63,72].

**Figure 3:**
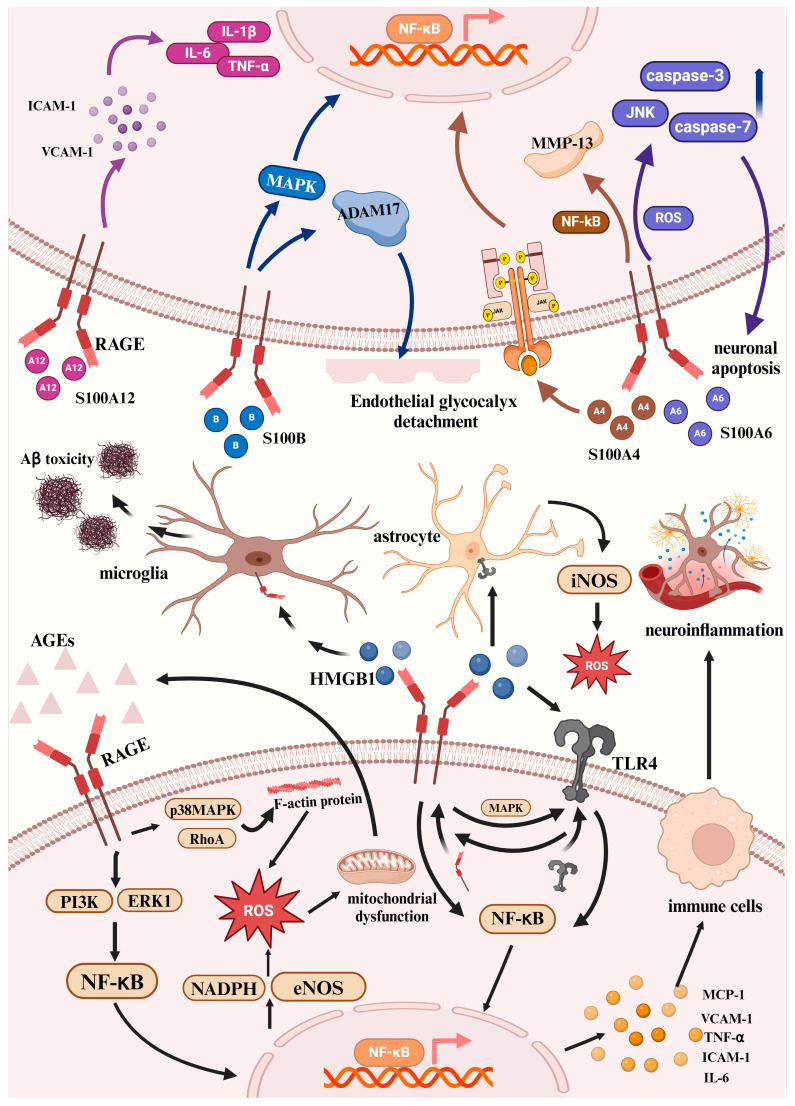
RAGE and its ligands interaction signaling pathways. Following the activation of the AGE-RAGE signaling cascade, the stimulation of the ERK1 and PI3K pathways can activate the nuclear factor NF-κB. This factor then translocates to the nucleus, promoting ROS production, which ultimately leads to cellular damage and mitochondrial dysfunction. NF-κB is capable of translocating to the nucleus, where it facilitates the transcription of pro-inflammatory cytokines and chemokines, including TNF-α, IL-6, MCP-1, VCAM-1, and ICAM-1. This process promotes inflammation and activates immune cells. AGEs can also activate p38 MAPK and RhoA kinase, which modifies the shape of the endothelial F-actin cytoskeleton and enhances the permeability of the endothelial cell monolayer. HMGB1 binding to RAGE acts as a key initiator of neuroinflammation and can also bind to TLR4, participating in neuroinflammation and neurotoxicity. The interaction between HMGB1 and RAGE initiates the activation of MAPK, which facilitates the translocation of TLR4 to the cell surface. Moreover, TLR4 at the cell membrane can interact with HMGB1, leading to the transcription and translation of RAGE, which in turn promotes the translocation of RAGE to the cell surface. HMGB1 inhibits microglial activation and phagocytosis through RAGE, promoting the accumulation of neuronal Aβ. HMGB1 activates astrocytes, increasing iNOS expression in cortical astrocytes via TLR4 signaling. S100A4 binds to RAGE and initiates a signaling cascade that ultimately causes MMP-13 release through the NF-κB pathway in chondrocytes. Two neurotrophic motifs on S100A4 activate the JAK/STAT pathway, preventing neurodegeneration. S100A6 promotes neuronal apoptosis through its interaction with RAGE, which triggers activation of JNK dependent on ROS as well as caspases-3 and -7. S100A12 binding to RAGE increases the activation and discharge of NF-κB, IL-1β, IL-6, and TNF-α. S100B accumulates at the RAGE receptor sites, resulting in the initiation of downstream MAPK pathways, which subsequently leads to the phosphorylation and activation of NF-κB. Stimulation of S100B/RAGE signaling can promote the shedding of the endothelial glycocalyx by enhancing the expression, translocation, and activity of the sheddase ADAM17 in endothelial cells. Abbreviations: RAGE, the receptor for advanced glycation end products; ERK, extracellular signal-regulated kinase; PI3K, phosphatidylinositol-3 kinase; ROS, reactive oxygen species; NF-κB, nuclear factor-kappa B; TNF-α, tumor necrosis factor-alpha; IL-6, interleukin-6; MCP-1, monocyte chemoattractant protein-1; VCAM-1, vascular cell adhesion molecule-1; ICAM-1, intercellular adhesion molecule-1; AGEs, advanced glycation end products; p38 MAPK, p38 mitogen-activated protein kinase; RhoA, Rho family GTPase A; HMGB1, high mobility group box 1; TLR4, toll-like receptor 4; MAPK, mitogen-activated protein kinase; Aβ, beta-amyloid peptide; iNOS, inducible nitric oxide synthase; MMP-13, matrix metalloproteinase-13; JAK/STAT, Janus kinase/signal transducer and activator of transcription; JNK, c-Jun N-terminal kinase; ADAM17, a disintegrin and metalloproteinase 17 (Created in https://BioRender.com). The arrow colors correspond to the colors of the ligands in the figure, each activating different signaling pathways (Accessed on 2 January 2025).

Following the activation of the AGE-RAGE signaling cascade, the stimulation of the ERK1 and phosphatidylinositol-3 kinase (PI3K) pathways can result in the initiation and movement of NF-κB into the nucleus. NF-κB facilitates the production of ROS, which ultimately contributes to cellular injury and mitochondrial dysfunction. Additionally, NF-κB is capable of translocating to the nucleus, where it initiates the expression of pro-inflammatory cytokines and chemokines like TNF-α, IL-6, monocyte chemoattractant protein-1 (MCP-1), VCAM-1, and intercellular adhesion molecule-1 (ICAM-1). This activity enhances inflammatory responses and activates immune cell activation (Figure 3). Activation of RAGE also intensifies the JAK/STAT signaling pathway and promotes the overexpression of genes related to the interferon response. The interactions between AGE and RAGE lead to the upregulation of NF-κB, which subsequently activates nitric oxide synthase (eNOS) and nicotinamide adenine dinucleotide phosphate (NADPH), resulting in increased production of ROS, enzyme activation, and further stimulation of NF-κB [4]. eNOS is essential for vasodilation and exerts antioxidative, antiproliferative, and antithrombotic effects when present at low intracellular concentrations. Decreased levels of eNOS lead to an increase in reactive oxygen and nitrogen species production, subsequently activating oxidative stress responses within cells [73].

Research indicates that AGEs initiate the activation of p38 MAPK and RhoA kinases, which subsequently modify the structure of the endothelial F-actin cytoskeleton and enhance the permeability of the endothelial monolayer [74]. The phosphorylation of threonine residues within myosin and ezrin/radixin/moesin (ERM) proteins results in changes to the cytoskeleton and compromises the integrity of the endothelial barrier. Alterations to the cytoskeleton, whether due to rearrangement or depolymerization, can lead to an increase in ROS production [75] (Figure 3). The p38 MAPK and JNK pathways can modulate apoptosis signals generated by RAGE. It is commonly accepted that, under glucose intolerance, increased levels of reducing carbohydrates inside cells—like glucose-6-phosphate, fructose, and fructose-3-phosphate—boost the production of AGEs, such as CML. This occurs because these reducing carbohydrates can enter the electron transport chain (ETC), resulting in dysfunction of the mitochondria and further enhancing AGE production. As levels of AGEs rise, there is also an increase in ROS production. This triggers the stimulation of protein kinase C (PKC) and activates the RAS, contributing to susceptibility to hypertension [76].

#### 4.1.3. AGE-RAGE Inhibitors

AGE inhibitors are primarily natural polyphenolic compounds or small synthetic molecules. These compounds can influence biological processes through multiple mechanisms, including serving as free radical scavengers or antioxidants, inhibiting AGE formation by sequestering 1,2-dicarbonyl compounds, or obstructing their effects. There is significant interest in creating inhibitors of AGEs as possible treatments for a range of age-related disorders. These conditions encompass diabetes and its associated neuropathies, along with neurodegenerative syndromes like AD, PD, TBI, and ALS.

Pyridoxamine (vitamin B6) acts as an inhibitor of AGEs and lipid peroxidation, thereby contributing to a reduction in AGE levels [77,78]. Pyridoxamine, because of its phenolic hydroxyl groups, functions as a free radical scavenger. This property allows it to sequester ROS and RNS, ultimately inhibiting oxidative stress and the formation of AGEs. Pyridoxamine diminishes the phosphorylation of Tau protein and the aggregation of β-tubulin caused by AGEs generated through the Maillard reaction involving glyceraldehyde. Additionally, it prevents functional impairments in neurite outgrowth induced by glyceraldehyde-derived AGEs, highlighting the potential therapeutic benefits of AGE inhibitor compounds [79]. In a mouse model exhibiting insulin resistance, pyridoxamine mitigated impaired sphingolipid metabolism linked to the accumulation of AGEs in the liver and its related AGE/RAGE signaling pathway. This effect was achieved by blocking the production of AGEs, which in turn alleviated insulin resistance.

Polyphenolic antioxidants prevent AGE formation by scavenging free radicals and chelating transition metal ions like Cu^2+^ and Fe^2+^. Additionally, they stimulate insulin signaling pathways and enhance glucose metabolism [80]. Phenolic aldehyde radicals achieve stabilization through resonance, rendering them steadier than ROS. As a result, polyphenolic agents can efficiently neutralize ROS. During this process, hydroxyl radicals are transformed into hydroxide anions, while phenolic compounds are converted into phenoxyl radicals. Polyphenols can also capture RNS, including peroxynitrite anions (ONOO^−^), via electrophilic or free radical aromatic nitration processes. Reactive nitrogen species and ROS are generated when transition metal ions, such as Cu^2+^ and Fe^2+^, interact with molecular oxygen, leading to the production of superoxide anion radicals (O^2−^). These radicals then participate in metal ion-catalyzed redox reactions, leading to the generation of highly reactive hydroxyl radicals (HO^−^). Superoxide anion radicals interact with nitric oxide (NO), a byproduct of nitric oxide synthase, resulting in the formation of the highly reactive nitrogen species ONOO^−^. By chelating metal ions, polyphenolic compounds effectively inhibit the generation of both ROS and reactive nitrogen species (RNS), which helps lower levels of AGEs.

Aminoguanidine is among the first AGE inhibitors to undergo clinical trials aimed at treating diabetes and its associated complications. Because of its strong reactivity in nucleophilic addition reactions, aminoguanidine rapidly reacts with 1,2-dicarbonyl compounds, including methylglyoxal, glyoxal, glucose ketone, and dehydroascorbic acid. 1,2-Dicarbonyl compounds display a high degree of electrophilicity and demonstrate greater reactivity compared to reducing sugars, including d-glucose, under physiological conditions. Therefore, capturing these intermediates prevents their further conversion to AGEs. In randomized, double-blind clinical studies involving 690 participants diagnosed with type 1 diabetes along with diabetic nephropathy and neuropathy, aminoguanidine provided clinical evidence indicating that inhibiting AGEs can improve outcomes associated with diabetic complications. However, these studies were discontinued due to adverse reactions observed in phase II/III clinical trials [81].

### 4.2. HMGB1

HMGB1 was initially identified as a pro-inflammatory cytokine and a late mediator in endotoxin-induced lethality. The HMGB1 protein consists of a single polypeptide chain containing 215 amino acids and features three distinct domains: the A-box (AA1–79), the B-box (AA89–162), and the acidic C-tail (AA186–215) [82]. Previous studies have shown that the A-box and B-box regions of HMGB1 are capable of binding to DNA, with the central functional region of the B-box being particularly significant [83]. Binding of the A-box can reduce the inflammatory response following acute inflammation damage.

HMGB1 is present in three isoforms: fully reduced HMGB1, sulfonylated HMGB1, and disulfide HMGB1. Among these, disulfide HMGB1 has been identified as the most potent variant in the context of inflammatory diseases. HMGB1 is extensively expressed across multiple organs and is primarily located in the cell nucleus [84], where it interacts with DNA or chromatin to regulate gene transcription and facilitate DNA repair mechanisms. Angiotensin II (ANG II) can enhance the expression of HMGB1, resulting in its acetylation. This process triggers the movement of HMGB1 from the nucleus to the cytoplasm and its dissociation from Sirtuin 1 (SIRT1), thereby facilitating M1 macrophage polarization. These findings highlight the crucial role of HMGB1 in maintaining inflammatory responses. HMGB1 can be released in a passive manner from necrotic cells or actively secreted by activated dendritic cells and macrophages, serving as a DAMP. This activity stimulates additional pro-inflammatory cascades and promotes the recruitment of immune cells [85].

#### 4.2.1. HMGB1-RAGE in AD

Research indicates that HMGB1 can trigger pro-inflammatory responses in various cell types, such as monocytes, neutrophils, endothelial cells, smooth muscle cells, and cancer cells, by interacting with RAGE [85,86]. HMGB1 can also mediate pro-inflammatory effects by interacting with other PRRs, including TLR2 and TLR4 [86]. In sterile inflammation related to ischemia–reperfusion injury (IRI), HMGB1 forms disulfide bonds and interacts with TLR-4, leading to enhanced synthesis of pro-inflammatory cytokines by macrophages [87]. Research indicates that HMGB1 may facilitate overlap and crosstalk between RAGE and TLRs. Specifically, HMGB1 attaches to RAGE receptors on the surface of cells, culminating in the activation of MAPK and promoting the translocation of TLR4 to the cell surface, while not influencing its transcription or translation [88]. Moreover, TLR4 found on the cell membrane can engage with HMGB1, resulting in the transcription and translation of RAGE. This process facilitates the relocation of RAGE to the cell membrane, allowing for further interactions with HMGB1 (Figure 3).

Data from various experimental models of AD suggest overexpression of HMGB1, RAGE, TLR4, NF-κB, and inflammatory mediators such as IL-1, IL-6, and TNF-α in hippocampal neurons. These results reinforce the notion that neuroinflammation plays a crucial role in AD and indicate that HMGB1 may mediate AD pathogenesis by activating the RAGE/TLR4 signaling pathway. Among the mediators involved in this neuroinflammatory process, HMGB1’s role as a chemotactic and pro-inflammatory factor is emphasized. It serves as a crucial initiator of neuroinflammation by directly binding to RAGE, contributing to neuroinflammation and Aβ accumulation, and interacts with TLR4 to participate in neuroinflammation and neurotoxicity [89] (Figure 3). Recent studies have detected increased levels of HMGB1 [90] in both the brains and cerebrospinal fluid of AD patients, as well as in mouse models of the condition [91,92,93]. Some in vitro research has suggested that the HMGB1-RAGE-TLR4 signaling pathway worsens hippocampal injury, contributing to memory impairment in patients with AD [94,95].

Moreover, HMGB1 hinders microglial activation and phagocytosis via RAGE, which facilitates the accumulation of neuronal Aβ [96]. From a mechanistic perspective, extracellular HMGB1 may function as a chaperone for Aβ, diminishing the clearance of Aβ by hindering microglial degradation of Aβ_40_ and the internalization of Aβ_42_ (Figure 3). Experiments have shown that HMGB1 injection impairs Aβ_42_ clearance in the ipsilateral hippocampus of rats and increases Aβ_42_-induced neurodegenerative lesions. Additionally, HMGB1 enhances Aβ-mediated neurotoxicity by inhibiting microglial phagocytosis and stabilizing Aβ monomers.

Another study found that the interaction between HMGB1 and TLR4 facilitates neuronal death under diabetic conditions [97]. HMGB1 stimulates astrocytes, which in turn boosts the secretion of pro-inflammatory cytokines and elevates the manifestation of iNOS in cortical astrocytes through TLR4 signaling (Figure 3). Moreover, it has been observed that the interaction between HMGB1, RAGE, and TLR4 intensifies Aβ accumulation, neuroinflammation, impairment of insulin signaling, and deficits in navigational memory. Mazarati [96] also found that overexpression of HMGB1 leads to memory deficits, potentially regulated by RAGE and TLR4. When mouse microglial cell line N9 was treated with trace amounts of Aβ, there was an upregulation of HMGB1, IL-1β, and the NLRP3 inflammasome. This implies the stimulation of microglia and the subsequent inflammatory reaction linked to AD. Analysis of HMGB1 levels in the cerebrospinal fluid of AD patients indicated that higher concentrations of HMGB1 correlate with accelerated dementia progression. This further implies that HMGB1 levels in cerebrospinal fluid could potentially serve as a biomarker for neurodegenerative advancement [98].

The role of HMGB1 largely relies on its location, the partners it binds to, and its redox state [99]. HMGB1 can be secreted into the extracellular environment by both immune and non-immune cell types in reaction to different stimuli, with its translocation generally indicating activation. Mild oxidation of HMGB1 results in the creation of disulfide bonds between Cys23 and Cys45, while Cys106 remains in a reduced state. This modification creates an active factor that stimulates the TLR4 receptor, transforming extracellular HMGB1 into a pro-inflammatory cytokine [100]. HMGB1 can stimulate the release of C-X-C motif chemokine ligand 12 (CXCL12) via RAGE. Similarly, following NF-κB activation [101], immune cells also produce CXCL12. This activation of NF-κB influences the regulation of various target genes, which includes pro-inflammatory cytokines like IL-1, IL-6, IL-8, and TNF-α, along with chemokines associated with NF-κB, like macrophage inflammatory protein 1 (MIP-1) and MCP-1. The majority of research indicates that the binding of HMGB1 to RAGE results in the direct activation of NF-κB, leading to the production of cytokines. However, some studies have indicated that when TLR4 is either functionally inactive or absent in macrophages that co-express TLR4 and RAGE, stimulation with any isoform of HMGB1 does not lead to cytokine production. This finding suggests that the HMGB1–RAGE interaction may need further examination to understand its role in direct cytokine activation. Recent research by Lu and Billiar [102] has revealed that RAGE facilitates a transport pathway for HMGB1, enabling its entry into the HMGB1 chaperone molecule complex through endocytosis. HMGB1 contains two separate binding sites for lipopolysaccharides (LPS), which allow it to transport LPS from the extracellular environment into the cytoplasm of caspase-11 via RAGE and lysosomal compartments [103]. Extracellular DNA that is associated with HMGB1 can be taken up through endocytosis via RAGE, allowing it to access homologous DNA receptors. These include endosomal TLR9 as well as cytoplasmic cGAS [104,105,106].

#### 4.2.2. HMGB1-RAGE Inhibitors

Research indicates that extracellular HMGB1 is integral to the development of chronic inflammation, implying that inhibitors targeting HMGB1 may help mitigate the risk of neurodegenerative changes. One such inhibitor is the anti-HMGB1 monoclonal antibody (m2G7), which attaches to an epitope found in box A, specifically within the amino acid sequence of HMGB1 from positions 53 to 63. This binding significantly influences the interactions of HMGB1 with RAGE and TLR4. The systemic inhibition of HMGB1 through the use of anti-HMGB1 monoclonal antibodies during the perioperative phase can avert postoperative cognitive dysfunction in aged rats [107].

Glycyrrhizin is well known for its anti-inflammatory effects as an inhibitor of HMGB1 and has been shown to reduce Alzheimer’s-like symptoms in mouse models of AD [108]. Glycyrrhizin prevents the movement of HMGB1 to the cytoplasm and reduces the levels of inflammatory cytokines [109]. In a model of experimental subarachnoid hemorrhage, resveratrol markedly reduced the activation of microglia as well as the expression levels of TLR4, HMGB1, MyD88, and NF-κB in the cerebral cortex [110]. Furthermore, resveratrol exhibited comparable neuroprotective and anti-inflammatory properties in a neonatal model of hypoxic–ischemic brain injury. Mechanistic investigations conducted both in vitro and in vivo have revealed that resveratrol activates SIRT1, leading to the attenuation of HMGB1/TLR4/MyD88/NF-κB signaling pathways and the subsequent reduction of neuroinflammation [111].

Metformin, an essential drug for managing type 2 diabetes, significantly contributes to preventing the translocation of nuclear HMGB1 into the cytoplasm, thereby maintaining its presence in the nucleus following cell activation. Additionally, metformin can directly bind to the C-terminal region of HMGB1, thereby downregulating inflammatory responses by inhibiting the extracellular activity of HMGB1 [112]. Moreover, this compound has the ability to suppress the release of HMGB1 and enhance the survival rate of mice experiencing endotoxemia [113].

The Nrf2/HO-1 pathway is also instrumental in regulating HMGB1 secretion. HO-1 functions to inhibit both the translocation and release of HMGB1 [114]. Nrf2 serves as a transcription factor sensitive to redox changes, overseeing the regulation of HO-1 expression [115].

Sirtuin 1 (SIRT1), an NAD+-dependent histone deacetylase, plays a direct role in reducing inflammation. SIRT1 is able to directly suppress the signaling pathways associated with HMGB1, improve metabolic and inflammatory responses, and maintain internal homeostasis. In response to inflammatory stimuli, HMGB1 undergoes extensive acetylation, which results in its release from the nucleus and eventual secretion into the extracellular space. Signaling pathways such as LPS and tumor necrosis factor-alpha promote the acetylation-driven release of HMGB1 from SIRT1. This process indirectly enhances the binding of HMGB1 to the protein Chromosome Region Maintenance 1 (CRM1), resulting in the translocation of HMGB1 [116,117]. SIRT1 engages with HMGB1 at various lysine residues located within the nuclear localization signal (NLS) region, facilitating the deacetylation of HMGB1. This action promotes its retention in the nucleus and diminishes its translocation to the cytoplasm [118].

Recombinant box A, functioning as an antagonist of HMGB1, has proven effective in treating multiple experimental models of both sterile and infectious tissue inflammation [119,120,121,122]. The underlying mechanism may involve box A interacting with RAGE, thereby competing with HMGB1 for binding to this receptor. This offers a compelling explanation for its function in inhibiting the RAGE-mediated endocytosis of HMGB1 chaperone molecule complexes, along with the subsequent activation of the immune response.

### 4.3. Aβ

Aβ peptides arise from the proteolytic cleavage of the transmembrane APP. This process initiates with the cleavage by β-secretase 1 (BACE1), which is then succeeded by further cleavage by γ-secretase [122]. The gradual buildup of Aβ in the brain is a key pathological characteristic of AD. During the initial stages of AD, the heightened accumulation of Aβ disrupts synaptic plasticity and contributes to the decline in memory function.

#### 4.3.1. Aβ-RAGE in AD

Studies have shown that in AD, RAGE expression is significantly elevated in areas with Aβ accumulation [123]. The binding of Aβ to RAGE demonstrates a strong affinity, mainly through its engagement with the V and C1 domains. The signaling pathways triggered by the interaction of Aβ with RAGE are essential for advancing the progression of AD. Research utilizing specific antibodies has revealed that Aβ oligomers associate with the V domain, whereas Aβ aggregates bind to the C1 domain [124,125]. Aβ_40_ and Aβ_42_ peptides are the main forms of Aβ in the brain, with Aβ_42_ being more closely associated with AD progression. Docking studies of RAGE with Aβ reveal that while RAGE and Aβ interactions exhibit some selectivity, Aβ_42_ can bind to nearly any site on the RAGE surface. The interaction regions can be categorized into two interfaces: Interface 1 encompasses residues 25–35, 57, 73, 77, 116–118, 123, 150, 186, and 216–221, situated between the V and C1 domains. Interface 2 consists of residues 198, 230, 233, 237, and 314, positioned between the C1 and C2 domains. Data from anatomical brain tissue studies, in vitro cell cultures, and transgenic (Tg) mouse models indicate that the interaction between Aβ and RAGE exacerbates Aβ accumulation, leading to neuronal stress, neuroinflammation, neuronal death, and impairments in learning and memory [126]. Pharmacological inhibition of RAGE signaling through specific antagonists TTP488 or FPSZM1 reduces neuroinflammation and amyloid deposition and improves behavioral performance in AD mouse models [49,50]. During the neuroinflammatory response in AD, some research indicates that Aβ interacts with surface toll-like receptors (TLRs) to stimulate microglia, resulting in the activation of the NF-κB signaling cascade. Consequently, this leads to the release of pro-inflammatory cytokines, which contribute to neurotoxicity and neurodegeneration [127].

Researchers developed a transgenic mouse model lacking RAGE (mAPP/RO mouse) [128] in an Aβ-rich environment, which is provided by a transgene encoding mutant APP/Aβ driven by a platelet-derived growth factor (PDGF) double-chain promoter [129]. It was first demonstrated that the brains of mAPP/RO mice show reduced amyloid pathology, with abnormal APP-Aβ metabolism suppressed by decreasing the activity of β- and γ-secretases, as evidenced by lower levels of C-terminal fragment beta (CTFβ) and C-terminal fragment gamma (CTFγ), and reduced levels of Beta-site amyloid precursor protein cleaving enzyme 1 (BACE1). Compared to 12-month-old mAPP mice, memory and learning impairments were attenuated. Importantly, compared to mAPP mice, mAPP/DN-RAGE mice expressing a neuronal signaling-deficient mutant RAGE showed reduced production of Aβ_40_ and Aβ_42_, as well as decreased β- and γ-secretase activity. These findings highlight that the RAGE-dependent signaling pathway regulates β- and γ-secretase cleavage of APP to produce Aβ, and that RAGE represents a potential therapeutic target for limiting abnormal APP-Aβ metabolism and preventing AD progression [8].

**Figure 4:**
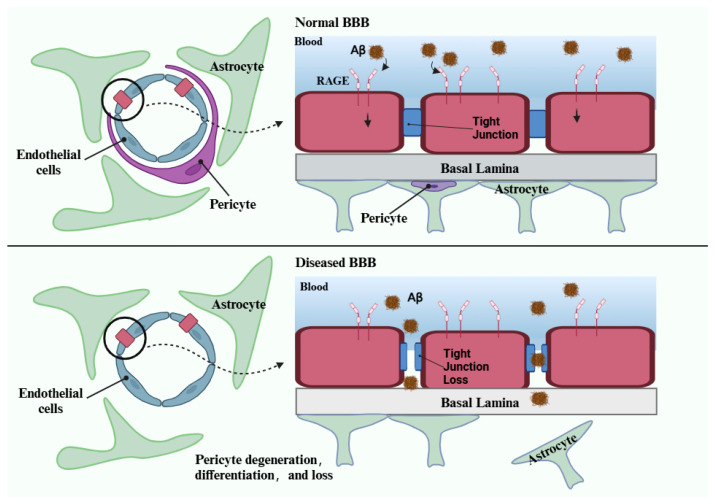
Aβ(Beta-amyloid peptide) transport across the normal and disrupted blood–brain barrier (BBB). RAGE mediates the transport of circulating Aβ from plasma to the brain across the BBB. Disruption of the BBB impairs the vascular clearance of brain Aβ and may increase the influx of peripheral Aβ into the brain. (Created in https://BioRender.com). (Accessed on 2 January 2025).

Studies indicate that early vascular conditions, especially damage to the BBB, are significant contributors to Aβ accumulation and neurodegeneration [48,130]. The blood–brain barrier (BBB) is a selective barrier between the blood circulation and the brain, formed by a monolayer of tightly sealed endothelial cells, pericytes, and astrocytic end-feet surrounding the blood vessels [131]. The vascular hypothesis of AD proposes that BBB disruption leads to the accumulation of various vasculotoxic and neurotoxic macromolecules in the brain, as well as reductions in cerebral blood flow (CBF) and hypoxia, thereby inducing functional and structural changes in neurons before Aβ deposition occurs [132,133]. Importantly, BBB disruption impairs the vascular clearance of brain Aβ and may increase the influx of peripheral Aβ into the brain, thereby elevating Aβ levels in the brain [132,134]. Reduced cerebral blood perfusion may also increase the expression and processing of APP, further promoting Aβ accumulation in the brain [135,136].In brain endothelial cells, RAGE mediates the transport of circulating Aβ from plasma to the brain across the BBB, which is an important factor in the pathogenesis of cerebral amyloid angiopathy [134]. Brain Aβ is removed through endocytosis mediated by the LRP1 receptor, which facilitates the clearance of brain-derived Aβ [137,138]. Aβ that is bound to RAGE is internalized across the endothelial cell membrane through endocytosis. The RAGE-mediated uptake of Aβ into the brain initiates neurovascular inflammation, which ultimately results in synaptic toxicity (Figure 4).

NLRP3 is pivotal in the pathophysiology of AD [139] by responding to cellular injury through the activation of caspase-1 [140]. This activation leads to the release of pro-inflammatory cytokines IL-1β and IL-18 [141], as well as the cleavage of gasdermin D (GSDMD), a critical protein involved in the pyroptosis cascade—a type of cell death initiated by pro-inflammatory signals. Thioredoxin-interacting protein (TXNIP) serves as a downstream signal of RAGE and has the ability to activate the NLRP3 inflammasome [142]. As an inhibitor of the ROS scavenger thioredoxin, TXNIP fosters oxidative stress, thereby connecting it to inflammation and contributing to cellular dysfunction. Studies [143] have demonstrated that the RAGE-TXNIP axis facilitates the movement of Aβ from the cell membrane to the mitochondria, leading to the abnormal activation of dynamin-related protein 1 (Drp1), a critical component involved in mitochondrial fission. This aberrant activation leads to mitochondrial dysfunction and subsequent activation of NLRP3, ultimately promoting the secretion of IL-1β and the activation of the pyroptosis-associated protein GSDMD.

#### 4.3.2. Aβ-RAGE Inhibitors

In recent years, various studies have focused on creating therapies designed to prevent Aβ from binding to RAGE, and identified RAGE inhibitors are regarded as promising options for disrupting the interaction between RAGE and Aβ. The peptide APDTKTQ (RP-1), which has high affinity for RAGE and significant homology with the Aβ peptide region 16–23 (KLVFFAED), is currently undergoing preclinical trials [144]. In SH-SY5Y cell cultures, RP-1 has been shown to prevent Aβ-induced cellular stress by binding to RAGE [145]. The RAGE antagonist peptide RAP, which is derived from the RAGE-binding domain of the HMGB1 protein, can block the RAGE signaling pathway and has been proven effective in acute lung inflammation, inhibiting Aβ peptide binding to RAGE [146] and reducing Aβ plaque burden and memory impairment in AD mouse [147] models.

Another promising therapeutic option for AD is FPS-ZM1, a potent blocker specifically targeting RAGE that inhibits the binding of Aβ to this receptor. Research has demonstrated that FPS-ZM1 can mitigate neurodegeneration and inflammation in APP (sw/0) transgenic mouse models of AD [148] and also prevent astrocyte-induced neuronal death in mice with the hSOD1G93A gene mutation.

Global docking studies were conducted to determine the binding sites of the inhibitors RAP, RP-1, and FPS-ZM1. The docking results indicate a distinct interaction interface between RAGE and RAP, where RAP predominantly associates with residues 116–125 and 179–183. These residues are part of a cross-region of RAGE that interacts with Aβ_42_. In contrast, the docking sites of RP-1 are distributed across the RAGE surface, with a high overlap in the Aβ interaction region. RAGE engages with FPS-ZM1 at residues 54, 96–98, and 114–120, which overlap with the Aβ_42_ interaction region, although complete overlap is not present. Therefore, RP-1 inhibitors show potential in mitigating the RAGE-Aβ interaction.

### 4.4. S100 Calcium-Binding Protein

S100 calcium-binding protein was initially identified in bovine brain extracts during the mid-1960s. This nervous system protein exhibits partial solubility in 100% saturated ammonium sulfate, which is the reason for its designation as S100 protein. The S100 protein family consists of acidic polypeptides with a low molecular weight, typically between 10 and 12 kDa. Since its initial discovery more than fifty years ago, the discovery of additional members within the S100 protein group has increased significantly. This family comprises 16 S100A proteins (S100A1 through S100A16), along with additional proteins including S100B, S100G, S100P, and S100Z [149]. S100 proteins are crucial in a variety of biological processes, such as inflammation, autoimmune disorders, and tumorigenesis [150,151,152].

Apart from the S100A8/S100A9 heterodimer, constituents of the S100 protein group show a notable extent of sequence and structural resemblance, typically forming homodimers [153]. Every S100 unit comprises four α-helices and contains two EF-hand domains, which are motifs characterized by a helix–loop–helix structure that serve as Ca^2+^ binding sites. These include a C-terminal canonical EF-hand comprising 12 amino acids and an N-terminal specific to the S100 family, which comprises 14 amino acids. The two EF-hand domains are linked through a loop or hinge region made up of 12–14 amino acids, which displays the highest degree of sequence variation among the family and is essential for interactions with target proteins.

S100 proteins engage with a range of target proteins and receptors, contributing to a wide array of functions including cell proliferation, differentiation, apoptosis, calcium homeostasis, energy metabolism, cytoskeletal regulation, and inflammation [153]. In this discussion, we concentrate on the physiological and pathological functions of S100A4, S100A6, S100A8, S100A9, S100A11, S100A12, and S100B in AD, as well as the possible mechanisms by which they may influence the progression of the disease.

#### 4.4.1. S100A4

The S100A4 protein, previously referred to by multiple names including mts1, 18A2, CAPL, FSP1, metastasin, p9Ka, PEL98, and calgranulin, has attracted growing interest in oncology due to its crucial role in enhancing cancer cell proliferation and metastasis. S100A4 is a key intracellular protein that modulates cell migration in physiological contexts [149]. When S100A4 levels are reduced in cells, macrophages in mice show a marked impairment in their ability to reach inflammation sites. Additionally, bone marrow-derived macrophages from these animals display decreased chemotactic response in vitro [154]. This suggests a close relationship between S100A4 and macrophage migration. Phagocytosis is a biological mechanism through which macrophages ingest and eliminate apoptotic cells that are undergoing apoptosis. Therefore, it is suggested that the depletion of S100A4 could influence the process of phagocytosis. Given that phagocytosis is essential for resolving inflammation and that neuroinflammation is a major factor in the development of AD, lower levels of S100A4 may hinder the effective resolution of inflammatory signals. From both inflammation and phagocytosis perspectives, S100A4 is related to the development of AD. Studies indicate that S100A4 may exert its effects by regulating the activity of specific cell receptors such as matrix metalloproteinase-13 (MMP-13) [155], matrix metalloproteinase-9 (MMP-9) [156], or RAGE [157,158].

The first description of the interaction between S100A4 and RAGE was in 2006 [159]. The authors revealed that S100A4 interacts with RAGE, triggering a signaling cascade that ultimately results in the release of MMP-13 through the NF-κB pathway in chondrocytes. This signaling pathway is inhibited by pretreatment with sRAGE or by advanced glycation end products that produce serum or dominant-negative RAGE structures. Two neurotrophic sequences found on S100A4 activate the Janus kinase/STAT pathway, contributing to the protection against neurodegeneration [160] (Figure 3). The Wnt/β-catenin signaling pathway is linked to cell migration facilitated by S100A4. A binding site for T-cell factor (TCF) is located in the 5′-untranslated region of the S100A4 promoter, where β-catenin has been demonstrated to bind. This interaction increases the expression of intracellular S100A4 protein, thereby enhancing migration and invasion in colon cancer cells [161]. Drugs such as calcimycin [162] and sulindac [163], which modulate the β-catenin pathway, have been isolated and may ultimately lead to reduced S100A4 expression. The transcription of S100A4 is directly mediated by the β-catenin/TCF complex [164]. Compounds such as calcimycin (a calcium ionophore), niclosamide (an antihelminthic agent), and sulindac (a nonsteroidal anti-inflammatory medication) are capable of promoting β-catenin degradation and/or preventing the assembly of the β-catenin/TCF complex, which in turn inhibits the transcription of S100A4. The covalent inhibitor 2,3-bis[2-hydroxyethylsulfanyl]-1,4-naphthoquinone, which targets S100A4 and S100B, also reduces the activity of several protein tyrosine phosphatases by altering the cysteine residue at the active site.

#### 4.4.2. S100A6

S100A6, a member of the EF-hand type Ca^2+^ binding protein family called S100, is known by several names, such as 2A9, 5B10, CABP, Cacy, calcium-binding protein, growth factor-inducible protein 2A9, and PRA (prolactin receptor-associated protein). S100A6 is found in neural progenitor cells situated in the dentate gyrus’s subgranular layer within the adult hippocampus, a key area for neurogenesis. Cells that express S100A6 are thought to serve as precursors to astrocytes. Research indicates that S100A6 is absent in mature astrocytes, implying that it may have a significant yet unclear function in the process of differentiating neural stem cells into astrocytes. It is possible that S100A6 must be downregulated to allow the differentiation of astrocyte precursors. S100A6 has also become a marker for glial precursor cells in neuroblastoma [165].

Studies have demonstrated that S100A6 is involved in the pathogenesis of AD, contributing to multiple biological processes associated with the disease. In murine models of AD, the S100A6 protein shows consistent upregulation in astrocytes within the white matter. Conversely, within the gray matter, nearly all S100A6 immunoreactivity is localized to astrocytes surrounding amyloid-β deposits.

Mechanistically, S100A6 promotes neuronal apoptosis by interacting with RAGE, which subsequently triggers activation of JNK driven by ROS as well as caspases 3 and 7 [166,167] (Figure 3). At the transcriptional level, the S100A6 gene promoter is activated by upstream transcription factors, including USF, NF-κB, Sp1, and Nrf2. S100A6 activates c-Jun NH2-terminal kinase [167], triggering apoptosis and ROS production, thus regulating RAGE-dependent survival in neuroblastoma cells.

Amlexanox downregulates S100A6 expression through an unknown mechanism, making MLL/AF4-positive acute lymphoblastic leukemia more sensitive to TNF-α treatment [168].

#### 4.4.3. S100A8/A9, S100A12

S100A8, S100A9, and S100A12 are structurally and functionally homologous and are frequently called S100/calcium-binding proteins (calprotectin). The S100 family of calcium-binding proteins can induce cytokine-like effects through their interaction with cell surface receptors, including RAGE and TLR-4 [152,169,170]. Ligands such as S100A8/A9 and S100A12 interact with RAGE, activating pathways within endothelial cells, smooth muscle cells (SMCs), and inflammatory processes. This activation stimulates the release of pro-inflammatory cytokines, which subsequently causes leukocyte infiltration, oxidative stress, and vascular inflammation, and aids in fibrous cap formation [171].

The stability and conformation of S100A8 and S100A9 proteins are significantly influenced by metal ion binding. Ca^2+^ binding induces structural changes that enable interactions with other proteins, while Zn^2+^ modulates their conformational characteristics. Upon interacting with these ions, S100A8/A9 heterodimers form stable heterotetrameric complexes in a manner dependent on both Ca^2+^ and Zn^2+^. In the S100A8/A9 heterotetramers, the binding sites are hidden, preventing interactions with RAGE and TLR-4, thus limiting their biological activity in inflammation [172]. Conversely, S100A8/A9 heterodimers exhibit a stronger affinity for RAGE and TLR-4, which in turn enhances inflammatory responses. Thus, targeting S100A8 and S100A9 inhibition may offer a successful approach to managing obesity-induced chronic inflammation by reducing the release of IL-1β and TLR-4 [173,174].

Although S100/calcium-binding proteins are considered to have pro-inflammatory effects, there are significant differences among S100A8, S100A9, and S100A12. For instance, the oxidation of methionine residues 63 and 83 on S100A9, along with the oxidation of cysteine 42 on S100A8, impairs the chemotactic and repellent actions of these proteins on neutrophils. In contrast, S100A12 exhibits greater resistance to oxidation and retains its biological activity even in conditions of heightened oxidative stress. Moreover, the polysaccharide enrichment of RAGE increases the binding affinity of S100A12 to RAGE by 30 times, highlighting S100A12’s potential to activate RAGE strongly.

S100A8 and S100A9 have been demonstrated to disturb the intestinal mucosal homeostasis, promoting the advancement of colitis by activating pro-inflammatory signaling pathways [175]. In AD patients, S100A9 expression is elevated in neurons [176]. Given that S100A9 possesses intrinsic amyloidogenic characteristics, its increased levels may enhance the synthesis and deposition of Aβ in the brain by activating neuroinflammatory pathways, thereby worsening neuroinflammation. Consequently, focusing on S100A9 during the prolonged inflammatory phase of Alzheimer’s disease could offer a potentially effective therapeutic strategy.

S100A12 is mainly found in the intracellular space of myeloid cells and functions as an inflammatory alarm signal when it is released. The expression of its gene diminishes as monocytes mature into macrophages. The expression of S100A12 protein significantly increases at inflammation sites, and its serum concentration may help predict the status of inflammatory diseases [150,177,178]. In vitro, S100A12 is capable of activating transmembrane proteins, including ICAM-1, VCAM-1, and RAGE analogs [152]. These proteins are crucial for the recruitment of leukocytes. When S100A12 interacts with RAGE, the pro-inflammatory response, observed in both in vivo and in vitro contexts, is marked by the activation and release of NF-κB, IL-1β, IL-6, and TNF-α (Figure 3).

A peptide system has been developed that inhibits two PRRs, incorporating inhibitory motifs for TLR4 and RAGE based on the sequences of S100A8 and S100A9 that interact with PRRs. This system is additionally enhanced by the inclusion of a CT peptide aimed at targeted delivery to the colon (rCT-S100A8/A9). The rCT-S100A8/A9 peptide system successfully blocks TLR4- and RAGE-mediated signaling in several mouse models of colitis, encompassing both acute and chronic colitis induced by dextran sulfate sodium (DSS) and colonic cancer triggered by azoxymethane (AOM)/DSS. Our findings indicate that rCT-S100A8/A9 can hinder the development of colitis and colorectal cancer by regulating the NLRP3 inflammasome activation. In 2016, Chang et al. [179] used heteronuclear single quantum coherence (HSQC) nuclear magnetic resonance experiments to elucidate the molecular interactions between the S100A9-RAGE V domain and S100A9-CHAPS. They presented a proposed model of a complex formed by Ca^2+^ binding mutants with CHAPS and the V domain of RAGE. CHAPS inhibited the interaction between S100A9 and the RAGE V domain, suggesting a potential new approach for drug development [179].

Chiou et al. performed two-dimensional HSQC NMR experiments to investigate the molecular mechanisms underlying S100A12 binding to RAGE [180]. Additionally, they discovered that tranilast, an allergy medication, inhibited the interaction between S100A12 and the V region of RAGE. Additionally, in the presence of Cu^2+^ and Zn^2+^ ions, quinoline derivatives such as Linomide, Tasquinimod, Paquinimod, Laquinimod, Tranilast, Amlexanox, Cromolyn, Methotrexate, and CHAPS have been shown to bind with S100A9 and S100A8/A9 complexes, preventing the attachment of S100A9 to RAGE. In vivo investigations demonstrated that Tasquinimod reduced the further release of TNF-α. Non-covalent antagonists, such as Paquinimod (ABR-215757), Tasquinimod (ABR-215050), and other quinoline-3-carboxamide derivatives, can interfere with the interactions of S100A8/S100A9 with TLR4 and RAGE.

#### 4.4.4. S100A11

S100A11, also referred to as S100C, is part of the S100 protein family and is regarded as a promoter of inflammatory processes. Increased expression of S100A11 has been noted in numerous inflammatory disorders, including infective endocarditis, idiopathic inflammatory myopathy, and rheumatoid arthritis [181]. Research has found [182] that S100A11, through its interaction with the multi-ligand receptor RAGE, activates the AKT/mTOR signaling pathway, thereby promoting lipid synthesis and exacerbating lipid deposition. As previously mentioned in the context of AD, one possible pathogenic factor is the inadequate supply of cholesterol to neurons. Cholesterol plays a crucial role in brain physiology, and changes in its concentration can result in abnormalities in Aβ metabolism and lead to neurodegenerative alterations. Therefore, the binding of S100A11 to RAGE may indirectly contribute to the development of AD.

#### 4.4.5. S100B

S100B is the predominant calcium-binding protein found in neural tissue, notably in astrocytes, and is widely present throughout the nervous system. It is regarded as one of the DAMPs released after initial or primary brain injury, contributing to secondary damage [183]. Studies have demonstrated that S100B is released into the extracellular environment by astrocytes, influencing these cells in an autocrine manner while affecting neurons in a paracrine manner [184,185].

The role of extracellular S100B (exS100B) is generally linked to the regulation of ROS generated by NOX as well as alterations in the levels and function of enzymes that control NO levels and reactivity [186,187]. NOX is crucial for the production of ROS mediated by RAGE. NOX-dependent ROS is strongly associated with damage to the hippocampus and cognitive decline, which may hasten the advancement of chronic neurodegenerative diseases. Therefore, the connection between exS100B and AD is also noteworthy.

The biological activity of exS100B is influenced by the specific cell type being stimulated and the local concentration of the exS100B protein. At physiological concentrations in the nanomolar range, exS100B typically exerts beneficial effects, supporting calcium (Ca^2+^) homeostasis, promoting cell differentiation, and facilitating tissue repair. Typically, micromolar levels of exS100B are linked to harmful processes, including inflammation, progression of melanoma, neurodegenerative disorders, neuroinflammation, aging, myocardial infarction, and disruptions in tissue repair. ExS100B at low concentrations exhibits neurotrophic effects; however, markedly increased levels of exS100B are associated with neuronal cell death. These effects are at least partly dependent on RAGE receptor activation [188,189]. At nanomolar levels, S100B has the capacity to enhance the production of the anti-apoptotic protein Bcl-2. Conversely, at micromolar amounts, S100B induces neuronal apoptosis through a mechanism that depends on RAGE. Low concentrations of exS100B stimulate a subtle pro-inflammatory response in astrocytes, promoting glial scar formation, whereas micromolar levels of exS100B transform astrocytes into a neurodegenerative phenotype.

Studies have shown that in the aging brain, S100B expression is elevated [190]. Astrocyte activation and impairment of the BBB contribute to a notable rise in S100B levels in serum as well as cerebrospinal fluid. Alterations in S100B regulation are progressively linked to several neurodegenerative disorders, such as AD and PD. For example, at low concentrations, S100B offers protection to neuroblastoma cells to protect Aβ-induced neurotoxicity through RAGE interaction. In contrast, at elevated concentrations, it exacerbates the neurotoxic effects caused by Aβ. By interacting with RAGE receptors, S100B serves as an extracellular modulator for a variety of cell types, including endothelial cells and astrocytes [191]. At the molecular scale, an overproduction of S100B accumulates at the sites of RAGE receptors, resulting in the activation of downstream MAPK. This process is subsequently followed by the phosphorylation and activation of NF-κB [192] (Figure 3). Within neurons, the S100B-RAGE complex is associated with neurite extension that depends on Cdc42-Rac1 signaling. In contrast, in mouse microglial cells and smooth muscle cells in blood vessels, this impact is mediated through the initiation of several effectors, including Src kinase.

Preclinical research suggests that S100B might have a time-sensitive role in the development of neurodegenerative diseases by modulating neuroinflammation and dopamine metabolism [193]. Elevated extracellular Ca^2+^ concentrations are linked to an enhanced formation of S100B oligomers, which may promote the stabilization of RAGE complexes or facilitate RAGE dimer formation. This process is crucial for RAGE-involved signaling pathways. The binding of S100B to RAGE can trigger the activation of small GTPases such as Ras, Rac1, and Cdc42. This activation subsequently triggers regulatory proteins like NF-κB and AP-1, resulting in an upregulation of COX-2, IL-1β, and TNF-α [194]. Moreover, the binding of S100B to RAGE has been demonstrated to trigger microglial activation and migration by engaging various signaling pathways, including Ras/Rac1-Cdc42/NF-κB, Ras/MEK/ERK1/2/NF-κB, Ras/Rac1-Cdc42/JNK/AP-1, and Src/Ras/PI3K/RhoA/ROCK [194]. At pro-inflammatory levels, S100B can promote the stimulation of microglia, causing the release of chemokines CCL3, CCL5, and CXCL12 via a mechanism dependent on RAGE.

Initiation of S100B/RAGE signaling can facilitate the release of the endothelial glycocalyx by increasing the expression, translocation, and operation of the key shedding enzyme a disintegrin and metalloproteinase 17 (ADAM17) in endothelial cells. Injury to the endothelial glycocalyx worsens BBB dysfunction and increases systemic vascular permeability [195], potentially leading to early cognitive decline and ultimately contributing to the development of AD [19] (Figure 3). S100B has been shown to stimulate intestinal glial cells, with its excessive expression linked to the onset and persistence of intestinal inflammation [196]. Growing evidence indicates that intestinal inflammation can alter the gut microbiota, with gut bacteria producing significant quantities of amyloid proteins and lipopolysaccharides. These substances could be involved in influencing signaling pathways and generating pro-inflammatory cytokines associated with the pathogenesis of AD [197].

Extensive studies reveal that extracellular S100 proteins are vital in the inflammatory responses linked to cancer, autoimmune disorders, and chronic inflammatory conditions [151,198]. S100 neutralizing antibodies could present a novel therapeutic approach. Within the S100 family, the development of small molecule inhibitors targeting S100B is the most progressed. Investigations of S100B inhibitor complexes have revealed three binding sites for small molecules. Site 1 serves as a binding target for inhibitors, such as the TRTK peptide or the C-terminal peptide of p53, and includes interactions with residues in the hinge region as well as helices 3 and 4. An example of an inhibitor for Site 1 is SEN205A. Site 2 is characterized by interactions with residues from both the hinge and helix 4, while Site 3 includes residues from the C-terminal loop and helix 1. Inhibitors focused on these sites comprise covalent modifications of S100B at Cys84 (site 2), as well as amlexanox and chlorpromazine [199], which attach to site 3 of S100A13 and S100B, respectively.

On the transcriptional level, duloxetine, an antidepressant that acts as a serotonin-norepinephrine reuptake inhibitor, has been recognized as a suppressor of S100B transcription [200]. Notably, duloxetine is capable of crossing the blood–brain barrier and has been shown to possess antitumor effects in vivo by reducing S100B levels in gliomas [200]. Intravenous delivery of arundic acid, a medication that inhibits the transcriptional biosynthesis of S100B in astrocytes, has demonstrated encouraging outcomes in models of ischemic brain injury. Administering arundic acid orally in the Tg2576 mouse model of Alzheimer’s disease has been found to enhance amyloid pathology, lessen Aβ deposition, and reduce glial cell proliferation [201].

## 5. Conclusions

The RAGE–ligand axis is closely associated with various diseases, where both RAGE and its ligands show significant upregulation and accumulation. RAGE is present in various cell types and engages with multiple ligands, creating a complex biochemical axis that is crucial in the pathogenesis of AD. This axis accelerates aging, promotes synaptic dysfunction and neuronal circuit impairment, and triggers mechanisms leading to Aβ and tau hyperphosphorylation through energy metabolism disturbances, mitochondrial dysfunction, oxidative stress, and inflammation.

RAGE plays a crucial role in the pathogenesis of AD by interacting with ligands such as AGEs, Aβ, HMGB1, and S100 proteins, accelerating the aging process and promoting neuroinflammation, synaptic dysfunction, amyloid accumulation, and cognitive decline. Inhibitors targeting RAGE and its ligands are considered emerging strategies for treating AD, with the potential to alleviate and treat this severe neurodegenerative disease. Existing studies support the efficacy of RAGE inhibitors in modulating inflammation and oxidative stress pathways associated with AD. However, their efficacy and safety in clinical applications still require further investigation. Future large-scale clinical studies should be conducted to evaluate the long-term efficacy and safety of RAGE inhibitors in AD patients, particularly in slowing cognitive decline and reducing amyloid accumulation. Additionally, further research is needed to explore the detailed molecular mechanisms of RAGE interactions with its ligands and how these interactions contribute to AD progression. A deeper understanding of these pathways could provide important clues for new therapeutic targets.
